# Supplementary material for: Bayesian refinement of protein structures and ensembles against SAXS data using molecular dynamics
Source: PLoS Comput Biol. 2017 Oct 18;13(10):e1005800. doi: 10.1371/journal.pcbi.1005800 (PMC5662244; doi:10.1371/journal.pcbi.1005800)
Supplement: S1 Archive — (BZ2) [file pcbi.1005800.s011.tar.bz2 › gromacs_4.62_bayesian_saxs_refi/share/html/online/g_anaeig.html]

g\_anaeig


| g\_anaeig Main Table of Contents |

**VERSION 4.6  
Sat 19 Jan 2013**

---

### Description

g\_anaeig analyzes eigenvectors. The eigenvectors can be of a
covariance matrix (g\_covar) or of a Normal Modes analysis
(g\_nmeig).

When a trajectory is projected on eigenvectors, all structures are
fitted to the structure in the eigenvector file, if present, otherwise
to the structure in the structure file. When no run input file is
supplied, periodicity will not be taken into account. Most analyses
are performed on eigenvectors -first to -last, but when
-first is set to -1 you will be prompted for a selection.

-comp: plot the vector components per atom of eigenvectors
-first to -last.

-rmsf: plot the RMS fluctuation per atom of eigenvectors
-first to -last (requires -eig).

-proj: calculate projections of a trajectory on eigenvectors
-first to -last.
The projections of a trajectory on the eigenvectors of its
covariance matrix are called principal components (pc's).
It is often useful to check the cosine content of the pc's,
since the pc's of random diffusion are cosines with the number
of periods equal to half the pc index.
The cosine content of the pc's can be calculated with the program
g\_analyze.

-2d: calculate a 2d projection of a trajectory on eigenvectors
-first and -last.

-3d: calculate a 3d projection of a trajectory on the first
three selected eigenvectors.

-filt: filter the trajectory to show only the motion along
eigenvectors -first to -last.

-extr: calculate the two extreme projections along a trajectory
on the average structure and interpolate -nframes frames
between them, or set your own extremes with -max. The
eigenvector -first will be written unless -first and
-last have been set explicitly, in which case all eigenvectors
will be written to separate files. Chain identifiers will be added
when writing a .pdb file with two or three structures (you
can use rasmol -nmrpdb to view such a .pdb file).

Overlap calculations between covariance analysis:  
**Note:** the analysis should use the same fitting structure

-over: calculate the subspace overlap of the eigenvectors in
file -v2 with eigenvectors -first to -last
in file -v.

-inpr: calculate a matrix of inner-products between
eigenvectors in files -v and -v2. All eigenvectors
of both files will be used unless -first and -last
have been set explicitly.

When -v, -eig, -v2 and -eig2 are given,
a single number for the overlap between the covariance matrices is
generated. The formulas are:  
difference = sqrt(tr((sqrt(M1) - sqrt(M2))^2))  
normalized overlap = 1 - difference/sqrt(tr(M1) + tr(M2))  
shape overlap = 1 - sqrt(tr((sqrt(M1/tr(M1)) - sqrt(M2/tr(M2)))^2))  
where M1 and M2 are the two covariance matrices and tr is the trace
of a matrix. The numbers are proportional to the overlap of the square
root of the fluctuations. The normalized overlap is the most useful
number, it is 1 for identical matrices and 0 when the sampled
subspaces are orthogonal.

When the -entropy flag is given an entropy estimate will be
computed based on the Quasiharmonic approach and based on
Schlitter's formula.

### Files

| option | filename | type | description |
| --- | --- | --- | --- |
| **-v** | eigenvec.trr | Input | Full precision trajectory: trr trj cpt |
| **-v2** | eigenvec2.trr | Input, Opt. | Full precision trajectory: trr trj cpt |
| **-f** | traj.xtc | Input, Opt. | Trajectory: xtc trr trj gro g96 pdb cpt |
| **-s** | topol.tpr | Input, Opt. | Structure+mass(db): tpr tpb tpa gro g96 pdb |
| **-n** | index.ndx | Input, Opt. | Index file |
| **-eig** | eigenval.xvg | Input, Opt. | xvgr/xmgr file |
| **-eig2** | eigenval2.xvg | Input, Opt. | xvgr/xmgr file |
| **-comp** | eigcomp.xvg | Output, Opt. | xvgr/xmgr file |
| **-rmsf** | eigrmsf.xvg | Output, Opt. | xvgr/xmgr file |
| **-proj** | proj.xvg | Output, Opt. | xvgr/xmgr file |
| **-2d** | 2dproj.xvg | Output, Opt. | xvgr/xmgr file |
| **-3d** | 3dproj.pdb | Output, Opt. | Structure file: gro g96 pdb etc. |
| **-filt** | filtered.xtc | Output, Opt. | Trajectory: xtc trr trj gro g96 pdb cpt |
| **-extr** | extreme.pdb | Output, Opt. | Trajectory: xtc trr trj gro g96 pdb cpt |
| **-over** | overlap.xvg | Output, Opt. | xvgr/xmgr file |
| **-inpr** | inprod.xpm | Output, Opt. | X PixMap compatible matrix file |

### Other options

| option | type | default | description |
| --- | --- | --- | --- |
| **-[no]h** | bool | no | Print help info and quit || **-[no]version** | bool | no | Print version info and quit || **-nice** | int | 19 | Set the nicelevel || **-b** | time | 0 | First frame (ps) to read from trajectory || **-e** | time | 0 | Last frame (ps) to read from trajectory || **-dt** | time | 0 | Only use frame when t MOD dt = first time (ps) || **-tu** | enum | ps | Time unit: fs, ps, ns, us, ms or s || **-[no]w** | bool | no | View output .xvg, .xpm, .eps and .pdb files || **-xvg** | enum | xmgrace | xvg plot formatting: xmgrace, xmgr or none || **-first** | int | 1 | First eigenvector for analysis (-1 is select) || **-last** | int | -1 | Last eigenvector for analysis (-1 is till the last) || **-skip** | int | 1 | Only analyse every nr-th frame || **-max** | real | 0 | Maximum for projection of the eigenvector on the average structure, max=0 gives the extremes || **-nframes** | int | 2 | Number of frames for the extremes output || **-[no]split** | bool | no | Split eigenvector projections where time is zero || **-[no]entropy** | bool | no | Compute entropy according to the Quasiharmonic formula or Schlitter's method. || **-temp** | real | 298.15 | Temperature for entropy calculations || **-nevskip** | int | 6 | Number of eigenvalues to skip when computing the entropy due to the quasi harmonic approximation. When you do a rotational and/or translational fit prior to the covariance analysis, you get 3 or 6 eigenvalues that are very close to zero, and which should not be taken into account when computing the entropy. |

---

http://www.gromacs.org  
gromacs@gromacs.org
